# Serum Soluble ST2 Correlated with Symptom Severity and Clinical Response of Sublingual Immunotherapy for House Dust Mite-Induced Allergic Rhinitis Patients

**DOI:** 10.1155/2021/5576596

**Published:** 2021-05-30

**Authors:** Kang Zhu, Cui Xia, Jingguo Chen, Chao Yu, Tianxi Gao, Jing Yan, Na Shao, Pin Zhu, Bin Sun, Xiaoyong Ren, Yanni Zhang

**Affiliations:** Department of Otolaryngology-Head and Neck Surgery, The Second Affiliated Hospital of Xi'an Jiaotong University, Xi'an, Shaanxi, China 710004

## Abstract

**Background:**

Suppressor of tumorigenicity 2 (ST2) is a key biomarker in inflammation and cardiovascular diseases, but limited data is available on its role in allergic rhinitis (AR).

**Objective:**

The aim of this study is to explore the role of serum soluble ST2 (sST2) in evaluating disease severity and predicting the efficacy of sublingual immunotherapy (SLIT) in house dust mite- (HDM-) induced AR patients.

**Methods:**

Eighty healthy controls (HC group) and 160 HDM-induced AR patients, including 40 mild patients (MAR group) and 120 moderate-severe patients (MSAR group), were recruited in this study. Serum was collected from all participants and levels of sST2 were determined by ELISA and the relationship between sST2 levels and disease severity was assessed. In the MSAR group, 109 patients received 3 years of SLIT, and the relationship between serum levels of sST2 and efficacy of SLIT was exampled.

**Results:**

Serum sST2 levels were increased in HDM-induced AR patients compared to the HC group (*P* < 0.001), and the concentrations were higher in the MSAR group than in the MAR group and HC group (all *P* < 0.05). Moreover, sST2 levels positively correlated with the total nasal symptom score (TNSS), visual analogue scale (VAS), and specific IgE levels (*P* < 0.05). Seventy-eight MSAR patients accomplished SLIT, and they were divided into an effective group (*n* = 40) and an ineffective group (*n* = 38). The serum sST2 levels in the effective group were lower than those in the ineffective group (*P* < 0.001). In addition, patients in the effective group levels exhibited significantly lower sST2 levels post-SLIT than pre-SLIT (*P* < 0.001), but no statistic difference was observed in the ineffective group (*P* > 0.05). Receiver operating characteristic (ROC) curve showed promising accuracy for predicting clinical efficacy of SLIT in AR patients (area under the curve = 0.839, *P* < 0.001).

**Conclusion:**

Serum sST2 is a potential biomarker for assessing disease severity and may serve as a sensitive biomarker for predicting the therapeutic response of SLIT in HDM-induced AR patients.

## 1. Introduction

Allergic rhinitis (AR) is an IgE-mediated type 1 hypersensitivity disease triggered by a spectrum of environmental allergens such as pollen, dust mites, cockroaches, and animal hair [1]. AR is one of the most common chronic illnesses with high prevalence all over the world, and house dust mites (HDMs) are the most prevalent allergen which induce perennial AR [2]. AR is a heterogeneous clinical disease with a wide degree of severity; many clinical variables, including total nasal symptom score (TNSS) and visual analogue scale (VAS), are utilized to evaluate its disease severity, but there is a lack of sensitive and specific variables or biological markers to reflect its disease activity [3, 4]. Therefore, exploring promising biomarkers to assess disease severity is extremely important to improve patient management and facilitate clinical research in AR. Currently, available therapeutic options for AR include patient education, allergen avoidance, pharmacotherapy, and allergen-specific immunotherapy (ASIT), and ASIT is the only etiological treatment for AR. Conventional AIT for AR comprises subcutaneous immunotherapy (SCIT) and sublingual immunotherapy (SLIT), and SLIT is more popular because of its convenience and good tolerability [5, 6]. Although SLIT is a safe and effective therapy, many AR patients still do not obtain a satisfactory therapeutic effect, and the rate of recovery fluctuates with a large range [7–9]. Treating patients who respond poorly to SLIT is futile and will lead to a waste of medical resources. Therefore, it is desirable to identify and exclude nonresponder patients before administrating SLIT. Although previous publications have found several potential indicators to objectively reflect the disease severity and predict the clinical response of SLIT, including serum-specific IgE, serum metabolites, and leukotriene A4 hydrolase [10–12], these biomarkers were not clinically available because of poor sensitivity and specificity. Thus, exploring biomarkers with high accuracy and reliability for monitoring disease severity and predicting the clinical efficacy of SLIT is extremely important.

Suppressor of tumorigenicity 2 (ST2) is a type 1 transmembrane protein encoded by the IL-1RL1 gene, and the released soluble form of ST2 (sST2) functions as an IL-33 receptor [13]. Previous studies have shown that sST2 played a crucial role in the regulation of immune and inflammatory response and was involved in several diseases [14, 15]. Magro and colleagues found that the serum sST2 levels were elevated in ulcerative colitis, and the levels positively correlated with the severity of colonic mucosal disease and inflammatory cytokines and might predict the therapeutic response of golimumab treatment [16]. Similarly, Chorin et al. ^17^ reported that sST2 levels were higher in the serum of systemic lupus erythematous (SLE) patients in comparison with healthy controls and the levels served a sensitive biomarker for evaluating disease severity and detecting subclinical diastolic dysfunction. Recent publications demonstrated that the increased serum sST2 levels could initiate and amplify Th2 inflammatory response and aggravate disease activity in asthma and food allergy [17–20]. Although sST2 presented important involvement in immune response in several diseases, its value as a serum biomarker has not yet been evaluated in HDM-induced AR patients. In this study, we sought to evaluate serum levels of sST2 as potential biomarkers for objectively assessing disease severity and predicting the efficacy of SLIT in HDM-induced AR patients.

## 2. Methods

### 2.1. Study Design and Participants

This was a single-center, prospective cohort study conducted to explore the role of serum soluble ST2 (sST2) in evaluating disease severity and predicting the efficacy of SLIT in patients with AR. The study followed the Declaration of Helsinki, and the study protocol was approved by the Human Ethical Committee of the Second Affiliated Hospital of Xi'an Jiaotong University. All recruited participants in this study provided written informed consent.

We consecutively recruited 160 adult patients with HDM-induced AR who visited our department during the period of January 2017 to June 2017. Eighty age- and sex-matched healthy volunteers without any allergic diseases were also enrolled as the healthy control (HC) group. All patients fulfilled the following inclusion criteria: meeting the diagnostic criteria provided by the allergic rhinitis and its impact on asthma (ARIA) guidelines [21]; medical history and allergic symptoms, such as sneezing, rhinorrhea, and nasal congestion, for at least three years; and a positive skin test to Dermatophagoides farinae and/or Dermatophagoides pteronyssinus (at least ++) and/or specific IgE (>0.35 IU/ml). Patients were excluded due to the following: other immunologic or inflammatory diseases; severe renal, liver, or heart dysfunction; age < 18 years; pregnancy or potential pregnancy; and systemic steroid or antiallergic treatment during the 4 weeks before enrollment. Clinical examination was performed, and demographic and clinical data were collected for all participants including age, sex, disease duration, body mass index (BMI), serum total and specific IgE, blood eosinophil, TNSS, and VAS.

### 2.2. Serum Sample Collection and sST2 Level Measurement

Participants scored their symptoms by utilizing the widely accepted TNSS which was a validated and easy-to-use patient-reported measure to subjectively delineate the clinical severity of AR [21, 22]. Accordingly, TNSS is the sum of 4 individual symptom scores for rhinorrhea, nasal congestion, nasal itching, and sneezing, each parameter ranged from 0 to 4. Based on TNSS, 160 HDM-induced AR patients were further categorized into mild (MAR) group (TNSS ≤ 4, *n* = 40) and moderate-severe (MSAR) groups (TNSS > 4, *n* = 120) referring to the ARIA criteria [21]. Moreover, we also evaluated the clinical activity of AR by a VAS, which was a 10-point scale from 0 to 10 (0 is not troubled and 10 is extremely troubled).

### 2.3. Immunotherapy

In the MSAR group, 109 patients were assigned to receive 3 years of standard SLIT. SLIT was conducted as previous studies described [10, 23]. Patients were administrated with standardized *Dermatophagoides farina* allergen drops purchased from Wolwo Pharma Biotechnology Company (Zhejiang, China) with the drops labeled from No.1 to No.5 containing proteins of 1, 10, 100, 333, and 1000 *μ*g/ml, respectively. The drug was self-administered by patients at the same time every day, kept under the tongue for 1-3 minutes, and then swallowed. The entire treatment included a dose escalation phase and a dose maintaining phase, and a 3-year maintenance was recommended to obtain long-term efficacy as previous studies recommended [24, 25]. Information regarding administration schedule was described in Table S1. During the SLIT, patient education and follow-up education were conducted to track and improve patient compliance.

### 2.4. Serum Sample Collection and sST2 Measurement

Blood samples were collected from each participant by 5 ml vacuum blood collection tubes without anticoagulation or coagulant. For the patients who were treated with SLIT, blood samples were collected pre-SLIT and 3 years post-SLIT. Blood samples were allowed to clot at room temperature for 60 minutes to separate out the red blood cells, then the tubes were centrifuged 4°C at 3500 rpm for 20 minutes, and the serum was collected and stored at -80°C for subsequent detection. Serum sST2 measurement was performed by an Enzyme-Linked Immunosorbent Assay- (ELISA-) utilizing human sST2 ELISA kit (Proteintech, Rosemont, USA) referring to the manufacturer's instructions. All serum samples were diluted at 1 : 40.

### 2.5. Data Collection and Outcome Assessment

During the treatment, all participants were followed up for at least 3 years and required to record their symptom and medication use. As SLIT cannot completely control allergic symptoms during the treatment especially when the primary symptoms are severe or the allergen load is heavy, adjunctive medication is prescribed including oral H1 antihistamine, intranasal corticosteroid, and intranasal antihistamine. The medication scores (MS) were evaluated as the sum of medication consumption for controlling AR symptoms over the previous week and recorded referring to the World Allergy Organization recommendations (1: oral or intranasal antihistamines, 2: nasal glucocorticoids, and 3: oral glucocorticoids) [26]. The symptom and medication score (SMS) was defined as the sum of TNSS and MS/7 as previously described [10, 27]. We followed the methods of Xie et al. [24] to evaluate the efficacy of SLIT on the basis of clinical symptom remission and the reduction in the consumption of combined pharmacologic therapy: “effective” was regarded as obtaining at least 30% reduction of SMS from the baseline level; otherwise, the SLIT was defined as ineffective.

### 2.6. Statistical Analysis

Statistical analysis was conducted with SPSS statistics software version 22.0 (IBM, Chicago, IL, USA), and figures were plotted with GraphPad Prism 7.0 (Software Inc., La Jolla, CA, USA). Continuous and categorical variables were displayed as mean ± standard deviation (SD) and number (%), respectively. Continuous variables were compared with one-way analysis of variance (ANOVA) or Kruskal–Wallis *H* test among three groups, and Student's *t* test or Mann–Whitney *U* was utilized between two groups. Chi-square test was performed in categorical variables. Spearman's correlation analysis was conducted to evaluate the correlation between TNSS, VAS, total and specific IgE levels, and sST2 levels. Receiver operating characteristic (ROC) curve was performed, and area under the curve (AUC), sensitivity, specificity, and cutoff were estimated. A *P* < 0.05 was accepted as statistical significance.

## 3. Results

### 3.1. Baseline Characteristics of Study Participants

The main demographic and clinical characteristics among three groups are shown in Table 1. There is no statistical difference in age, sex, and BMI among three groups, and disease duration between the MSAR group and the MAR group (*P* > 0.05). In the MSAR group, the total IgE, HDM-specific IgE, blood eosinophil, TNSS, and VAS were higher than those in the MAR and HC groups (all *P* < 0.001).

### 3.2. sST2 Levels Elevated in AR Patients and Correlated with Clinical Variables

**Figure 1:**
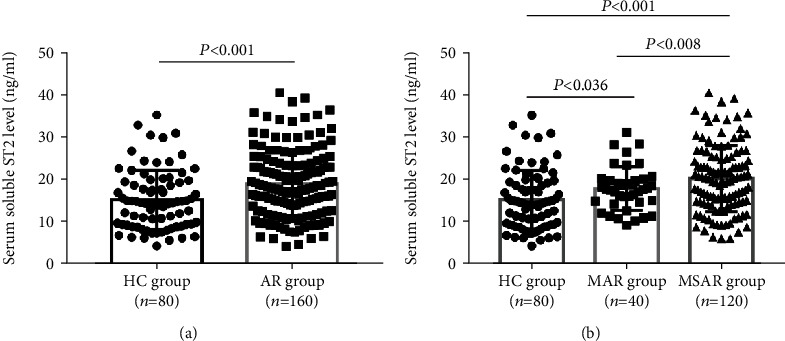
Serum sST2 concentrations were increased in AR patients than in healthy controls. (a) sST2 levels were elevated in the AR group in comparison with the HC group; (b) the serum sST2 levels were higher in the MSAR group than in the MAR and the HC group. sST2: soluble suppressor of tumorigenicity 2; AR: allergic rhinitis; HC: healthy control; MAR: mild allergic rhinitis; MSAR: moderate-severe allergic rhinitis.

**Figure 2:**
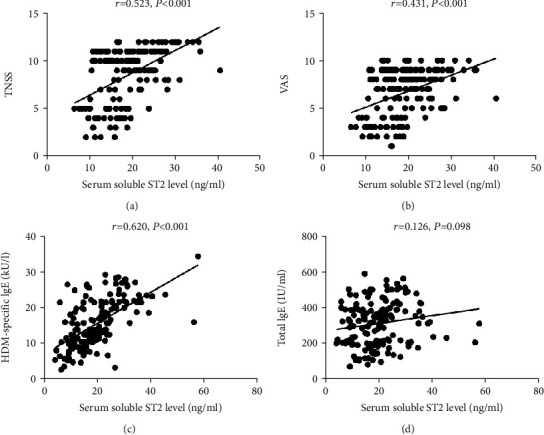
Correlation between serum sST2 levels and TNSS (a), VAS (b), HDM-specific IgE (c), and total IgE (d). sST2: soluble suppressor of tumorigenicity 2; HDM: house dust mite; TNSS: total nasal symptom score; VAS: visual analogue scale.

Figure 1(a) exhibited the comparison of sST2 levels between the AR group and the HC group. Serum sST2 levels were 19.8 ± 7.5 ng/ml in HDM-induced AR patients, which were higher than healthy controls (14.3 ± 6.9 ng/ml, *P* < 0.001). Moreover, the sST2 levels were significantly elevated in the MSAR group (21.3 ± 7.1 ng/ml) and the MAR group (18.8 ± 5.1 ng/ml) in comparison with the HC group (14.3 ± 6.9 ng/ml, *P* < 0.05) (Figure 1(b)). According to Spearman's correlation analysis results, the elevated sST2 concentrations positively correlated with TNSS (*r* = 0.523, *P* < 0.001), VAS (*r* = 0.431, *P* < 0.001), and HDM-specific IgE (*r* = 0.620, *P* < 0.001) (Figure 2).

### 3.3. sST2 Levels Acted as a Potential Biomarker for Predicting Efficacy of SLIT

**Figure 3:**
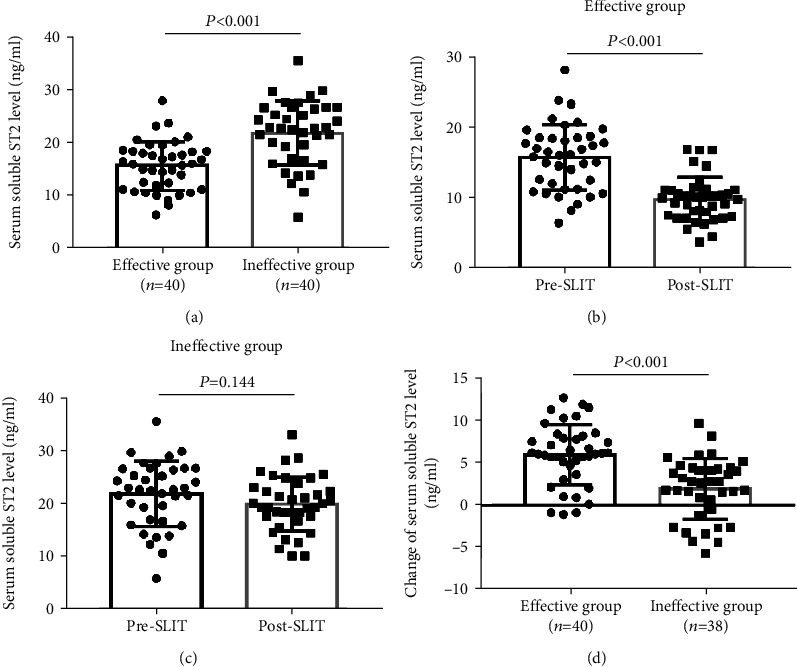
The serum levels sST2 in the effective group and ineffective group. (a) The sST2 levels were lower in the effective group than in the ineffective group; (b) patients in the effective group exhibited significantly lower sST2 levels post-SLIT than pre-SLIT; (c) no statistical difference was observed in the ineffective group between post-SLIT and pre-SLIT. (d) the change of sST2 levels was higher in the effective group than in the ineffective group. sST2: soluble suppressor of tumorigenicity 2; SLIT: sublingual immunotherapy.

**Figure 4:**
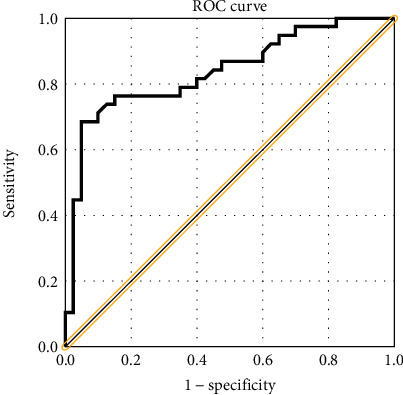
ROC curve analysis of serum sST2 in predicting the efficacy of SLIT. ROC: receiver operating characteristic; sST2: soluble suppressor of tumorigenicity 2; SLIT: sublingual immunotherapy.

In this study, 78 MSAR patients finally finished the 3-year SLIT and provided complete follow-up data. Among these patients, 40 patients were grouped into the effective group and the other 38 cases were grouped into the ineffective group; the overall effective rate was 51.3%. During the follow-up, 6 patients reported adverse reactions, including pruritus and swelling of the mouth and tongue in 4 patients and aggravating rhinitis in 2 patients; no severe systemic adverse reaction was reported.  Table 2 showed that no statistical difference was observed in age, sex, BMI, disease duration, total IgE, blood eosinophil, TNSS, and VAS between two groups (*P* > 0.05), but HDM-specific IgE levels were lower in the effective group than in the ineffective group (*P* = 0.003). The serum levels of sST2 were significantly lower in the effective group than in the ineffective group (16.8 ± 3.4 ng/ml vs. 23.7 ± 7.2 ng/ml, *P* < 0.001) (Figure 3(a)). In addition, Figures 3(b) and 3(c) showed that patients in the effective group exhibited significantly lower sST2 levels post-SLIT than pre-SLIT (*P* < 0.001), but no statistical difference was observed in the ineffective group (*P* > 0.05). In the effective group, the change of sST2 levels was 6.0 ± 4.1 ng/ml, which were higher than 1.9 ± 3.4 ng/ml in the ineffective group (*P* < 0.001) (Figure 3(d)). The ROC curve exhibited promising accuracy for predicting clinical efficacy of SLIT in AR patients (area under the curve = 0.839, *P* < 0.001), and the cutoff value with optical sensitivity and specificity was 20.4 ng/ml (sensitivity = 0.950, specificity = 0.634) (Figure 4).

## 4. Discussion

In the present study, we showed that serum sST2 levels were increased in HDM-induced AR patients especially in MSAR patients in comparison with healthy controls, and the elevated sST2 levels positively correlated with TNSS, VAS, and HDM-specific IgE. Moreover, in the subgroup of MSAR patients who received SLIT, the concentrations of sST2 were significantly lower in the effective group than in the ineffective group, and the levels were decreased in the samples collected post-SLIT than pre-SLIT, but no statistic difference was observed in the ineffective group. ROC results demonstrated promising reliability and accuracy in predicting the therapeutic efficacy of SLIT. Taken together, our observations indicated that serum sST2 might serve as a potential biomarker for objectively reflecting the disease severity and predicting the clinical efficacy of SLIT in HDM-induced AR patients.

ST2 functioning as a decoy receptor for IL-33 has been observed in several cells, such as mast cells, macrophages, dendritic cells, eosinophils, and group 2 innate lymphoid cells, and was pivotal in the immune response and inflammatory cytokine production [28–30]. Previous publications have demonstrated that the IL-33/ST2 signaling axis was involved in the occurrence and development of inflammatory and allergic diseases including rheumatoid arthritis, inflammatory bowel disease, SLE, food allergy, and asthma [20, 31–33]. Zheng et al. ^32^ observed that elevated ST2 activated the myeloid dendritic cells and promoted CD4+ T cells toward Th2 differentiation inducing the secretion of Th2-type cytokines (IL-5 and IL-13) and eosinophilic inflammation in nasal polyps. A recent study found that the serum levels of sST2 were increased in the patients with active SLE, and the elevated sST2 might induce the Th1 to Th2 shift of the immune reactions and aggravated the disease activity and tissue and organ damage [17, 34]. However, in another study, the authors showed that the levels of sST2 negatively correlated with fractional exhaled nitric oxide, and they speculated that sST2 might exhibit a potential protective effect on the development of eosinophilic airway inflammation in asthmatic children [20]. Although growing evidence has shown that sST2 is upregulated in allergic disorders, the role of sST2 in AR was unclear.

In this study, we demonstrated that serum sST2 concentrations were increased in AR patients especially in cases with MSAR and the elevated sST2 associated with the clinical severity recorded by symptom score scales. Our observations were in line with previous results in other allergic diseases such as food allergy, asthma, and atopic dermatitis [20, 30, 35]. ST2 was a key mediator regulating the functions of immune cells and was involved in the antigen-presenting process and promoting the immune response [15, 32]. When patients were exposed to an allergic environment, the allergens will stimulate the endothelial cells and epithelial cells and predominantly promote the secretion of IL-33, the increased levels of IL-33 will upregulate the expression of ST2 especially in the dendritic cells, the activated dendritic cells will enhance CD4+ T differentiation to Th2 cells and induce the production of Th2-type cytokines (IL-4, IL-5, and IL-13), and the high concentrations of these cytokines will cause the activation of B cells and secretion of IgE and enhance the mast cell degranulation and histamine release, resulting in exacerbation of allergic symptoms in AR patients [36–38]. Therefore, we have reasons to believe that sST2 is involved in the occurrence and development of AR, but the underlying mechanism needs to be discovered in further experimental studies.

SLIT has been proven to be effective and safe in AR patients for many years [5, 7]. Although previous studies demonstrated that SLIT significantly improved the allergic symptoms and decreased the consumption of rescue medication in AR patients, it still did not improve the disease status in a certain proportion of patients after a long duration of treatment [8, 27]. How to predict the treatment response before the onset of SLIT is a thorny problem that all patients and allergy specialists have to confront. Currently, there is no established modality to predict clinical efficacy of SLIT that can guide the clinical practice. In the current study, we firstly found that the serum sST2 levels in the effective group were decreased compared to those in the ineffective group; patients who obtained effective treatment exhibited significantly lower sST2 levels in comparison with baseline levels. In addition, ROC results showed that serum sST2 was a reliable and accurate biomarker to predict the efficacy of SLIT. Further evidence has indicated that ST2 was a pivotal molecule in regulating functional dendritic cell subsets in the antigen presentation process to affect Th2 inflammatory response, activation of B cells, and secretion and mast cell degranulation [15, 30, 39]. As SLIT was a treatment that induced immune tolerance, repetitive stimulation of a specific antigen might affect the function of dendritic cells and interdict the antigen presentation process and initiation of immune response [26]. Combined with our findings, we can speculate that sST2 might act as a crucial biomarker in the underlying mechanism of SLIT in HDM-induced AR patients, and sST2 could be clinically meaningful as an objective biomarker to predict the clinical efficacy of SLIT.

Several limitations exist in the current study. First, the numbers of recruited participants are relatively small, and a validation cohort study is required to strengthen the conclusions. Second, all the recruited participants are from a single center with the same ethnicity and region, which might increase the risk of selection bias. Third, we did not evaluate the change of serum ST2 levels at 1-year follow-up when some patients responded to SLIT. Last, there is a lack of unified criteria in assessing the efficacy of SLIT, and this may limit the applicability of obtained results in a clinical way.

## 5. Conclusion

This prospective study demonstrated that the sST2 levels in the serum of HDM-induced AR patients were elevated and associated with a clinical symptom score, suggesting serum sST2 may serve as an objective indication to evaluate the disease severity of AR. We also found that serum sST2 may be a surrogate biomarker in predicting the therapeutic response of SLIT in HDM-induced AR patients. These results may provide novel insights into pathophysiological mechanisms of HDM-induced AR and contribute to discover the underlying mechanisms of SLIT.

## Figures and Tables

**Table 1:** The demographic and clinical characteristics of participants.

Variables	HC (*n* = 80)	MAR (*n* = 40)	MSAR (*n* = 120)	*P* value
Age (year)	28.6 ± 7.3	28.4 ± 9.1	30.6 ± 10.4	0.157
Sex, male (%)	39 (48.8)	23 (57.5)	63 (52.5)	0.659
Disease duration (year)	NA	4.1 ± 2.0	4.5 ± 1.8	0.249
BMI (kg/m^2^)	22.6 ± 1.7	22.6 ± 1.9	22.3 ± 1.6	0.813
Total IgE (IU/ml)	49.3 ± 16.9	166.1 ± 69.8	360.1 ± 156.4	<0.001
HDM specific IgE (kU/l)	0.2 ± 0.1	15.8 ± 4.1	22.3 ± 7.9	<0.001
Blood eosinophil (n/*μ*l)	138.6 ± 49.7	294.5 ± 124.6	370.8 ± 164.7	<0.001
TNSS	1.4 ± 0.5	4.2 ± 1.0	10.0 ± 2.3	<0.001
VAS	1.0 ± 0.4	3.0 ± 0.6	7.9 ± 1.9	<0.001

HC: healthy control; MAR: mild allergic rhinitis; MSAR: moderate-severe allergic rhinitis; BMI: body mass index; HDM: house dust mite; TNSS: total nasal symptom score; VAS: visual analogue scale; NA: not applicable.

**Table 2:** The demographic and clinical variables between two groups.

Variables	Effective group (*n* = 40)	Ineffective group (*n* = 38)	*P* value
Age (year)	30.6 ± 9.1	31.6 ± 9.1	0.610
Sex, male (%)	22 (55.0)	18 (47.4)	0.651
Disease duration (year)	4.8 ± 1.8	4.4 ± 1.6	0.268
BMI (kg/m^2^)	22.4 ± 1.7	22.0 ± 1.2	0.247
Total IgE (IU/ml)	296.1 ± 158.9	343.4 ± 172.5	0.211
HDM specific IgE (kU/l)	18.9 ± 6.7	23.8 ± 8.2	0.003
Blood eosinophil (n/*μ*l)	314.5 ± 124.6	370.8 ± 164.7	0.092
TNSS	9.1 ± 3.2	9.7 ± 2.9	0.389
VAS	7.1 ± 2.2	7.8 ± 2.5	0.193

BMI: body mass index; HDM: house dust mite; TNSS: total nasal symptom score; VAS: visual analogue scale.

## Data Availability

The data used to support the observations of this study are available from the corresponding author upon request.

## Supplementary Materials

Supplementary Materials Information regarding allergen administration schedule in AR patients is described in Table S1.

## References

[1] Bousquet J., Anto J. M., Bachert C. (2020). Allergic rhinitis. *Nature Reviews. Disease Primers*.

[2] Meng Y., Wang C., Zhang L. (2019). Recent developments and highlights in allergic rhinitis. *Allergy*.

[3] Meng Y., Wang C., Zhang L. (2020). Advances and novel developments in allergic rhinitis. *Allergy*.

[4] Okubo K., Kurono Y., Ichimura K. (2020). Japanese guidelines for allergic rhinitis 2020. *Allergology International*.

[5] Drazdauskaitė G., Layhadi J. A., Shamji M. H. (2020). Mechanisms of allergen immunotherapy in allergic rhinitis. *Current Allergy and Asthma Reports*.

[6] Lam H. Y., Tergaonkar V., Ahn K. S. (2020). Mechanisms of allergen-specific immunotherapy for allergic rhinitis and food allergies. *Bioscience Reports*.

[7] Chinese Society of Allergy (CSA), Li H., Chen S. (2019). Chinese guideline on sublingual immunotherapy for allergic rhinitis and asthma. *Journal of Thoracic Disease*.

[8] Licari A., Castagnoli R., Brambilla I. (2018). Biomarkers of immunotherapy response in patients with allergic rhinitis. *Expert Review of Clinical Immunology*.

[9] Zissler U. M., Schmidt-Weber C. B. (2020). Predicting success of allergen-specific immunotherapy. *Frontiers in Immunology*.

[10] Xie S., Jiang S., Zhang H. (2021). Prediction of sublingual immunotherapy efficacy in allergic rhinitis by serum metabolomics analysis. *International Immunopharmacology*.

[11] Liu W., Zeng Q., Luo R. (2020). Predictors for short-term efficacy of allergen-specific sublingual immunotherapy in children with allergic rhinitis. *Mediators of Inflammation*.

[12] Ma T. T., Cao M. D., Yu R. L. (2020). Leukotriene A4 hydrolase is a candidate predictive biomarker for successful allergen immunotherapy. *Frontiers in Immunology*.

[13] Zhao Q., Men L., Li X. M. (2019). Circulating mif levels predict clinical outcomes in patients with st-elevation myocardial infarction after percutaneous coronary intervention. *The Canadian journal of cardiology.*.

[14] Lobdell K. W., Parker D. M., Likosky D. S. (2018). Preoperative serum st2 level predicts acute kidney injury after adult cardiac surgery. *The Journal of Thoracic and Cardiovascular Surgery*.

[15] Krzystek-Korpacka M., Kempiński R., Bromke M., Neubauer K. (2020). Biochemical biomarkers of mucosal healing for inflammatory bowel disease in adults. *Diagnostics (Basel, Switzerland)*.

[16] Magro F., Lopes S., Silva M. (2019). Soluble human suppression of tumorigenicity 2 is associated with endoscopic activity in patients with moderate-to-severe ulcerative colitis treated with golimumab. *Therapeutic Advances in Gastroenterology*.

[17] Mok M. Y., Huang F. P., Ip W. K. (2010). Serum levels of IL-33 and soluble ST2 and their association with disease activity in systemic lupus erythematosus. *Rheumatology (Oxford, England)*.

[18] Zoltowska A. M., Lei Y., Fuchs B., Rask C., Adner M., Nilsson G. P. (2016). The interleukin-33 receptor ST2 is important for the development of peripheral airway hyperresponsiveness and inflammation in a house dust mite mouse model of asthma. *Clinical and Experimental Allergy*.

[19] Pusceddu I., Dieplinger B., Mueller T. (2019). ST2 and the ST2/IL-33 signalling pathway-biochemistry and pathophysiology in animal models and humans. *Clinica chimica acta; international journal of clinical chemistry*.

[20] Ketelaar M. E., Westerlaken-van Ginkel C. D., Nawijn M. C., Ej Dubois A., Koppelman G. H. (2021). IL-1RL1a serum levels and IL1RL1 SNPs in the prediction of food allergy. *Clinical and Experimental Allergy*.

[21] Brożek J. L., Bousquet J., Agache I. (2017). Allergic rhinitis and its impact on asthma (ARIA) guidelines--2016 revision. *The Journal of Allergy and Clinical Immunology*.

[22] Adamko D. J., Khamis M. M., Steacy L. M., Regush S., Bryce R., Ellis A. K. (2018). Severity of allergic rhinitis assessed by using urine metabolomic profiling: proof of concept. *The Journal of Allergy and Clinical Immunology*.

[23] Gao Y., Lin X., Ma J., Wei X., Wang Q., Wang M. (2020). Enhanced efficacy of dust mite sublingual immunotherapy in low-response allergic rhinitis patients after dose increment at 6 months: a prospective study. *International Archives of Allergy and Immunology*.

[24] Xie S., Zhang H., Wang F. (2020). Activated leukocyte cell adhesion molecule as a biomarker for disease severity and efficacy of sublingual immunotherapy in allergic rhinitis. *International Immunopharmacology*.

[25] Chinese Society of Allergy (CSA) and Chinese Allergic Rhinitis Collaborative Research Group (C2AR2G), Bao Y., Chen J. (2017). Chinese guideline on allergen immunotherapy for allergic rhinitis. *Journal of Thoracic Disease*.

[26] Canonica G. W., Baena-Cagnani C. E., Bousquet J. (2007). Recommendations for standardization of clinical trials with allergen specific immunotherapy for respiratory allergy. A statement of a world allergy organization (WAO) taskforce. *Allergy*.

[27] Liu Z., Lu H., Feng X., Hu L., Wang J., Yu H. (2020). Predictive methods for efficacy of house dust mite subcutaneous immunotherapy in allergic rhinitis patients: a prospective study in a Chinese population. *International forum of allergy & rhinology.*.

[28] Liu G., Liu F. (2020). Advances of IL-33/ST2 signaling pathway in allergic rhinitis. *Lin chuang er bi yan hou tou jing wai ke za zhi = Journal of clinical otorhinolaryngology, head, and neck surgery*.

[29] Chen W. Y., Tsai T. H., Yang J. L., Li L. C. (2018). Therapeutic strategies for targeting IL-33/ST2 signalling for the treatment of inflammatory diseases. *Cellular Physiology and Biochemistry: International Journal of Experimental Cellular Physiology, Biochemistry, and Pharmacology*.

[30] Takatori H., Makita S., Ito T., Matsuki A., Nakajima H. (2018). Regulatory mechanisms of IL-33-ST2-mediated allergic inflammation. *Frontiers in Immunology*.

[31] Zheng R., Chen Y., Shi J. (2020). Combinatorial IL-17RB, ST2, and TSLPR signaling in dendritic cells of patients with allergic rhinitis. *Frontiers in Cell and Development Biology*.

[32] Huang R., Mao W., Wang G. (2020). Synergistic relationship between TSLP and IL-33/ST2 signaling pathways in allergic rhinitis and the effects of hypoxia. *International forum of allergy & rhinology.*.

[33] Ishikawa S., Shimizu M., Ueno K., Sugimoto N., Yachie A. (2013). Soluble ST2 as a marker of disease activity in systemic juvenile idiopathic arthritis. *Cytokine*.

[34] Chorin E., Hochstadt A., Arad U. (2020). Soluble ST2 and CXCL-10 may serve as biomarkers of subclinical diastolic dysfunction in SLE and correlate with disease activity and damage. *Lupus*.

[35] Dijk F. N., Xu C., Melén E. (2018). Genetic regulation of IL1RL1 methylation and IL1RL1-a protein levels in asthma. *The European respiratory journal.*.

[36] Bui T. T., Piao C. H., Song C. H., Chai O. H. (2017). Skullcapflavone II attenuates ovalbumin-induced allergic rhinitis through the blocking of Th2 cytokine production and mast cell histamine release. *International Immunopharmacology*.

[37] Qin X., Liu M., Zhang S., Wang C., Zhang T. (2019). The role of IL-36*γ* and its regulation in eosinophilic inflammation in allergic rhinitis. *Cytokine*.

[38] Liao B., Cao P. P., Zeng M. (2015). Interaction of thymic stromal lymphopoietin, IL-33, and their receptors in epithelial cells in eosinophilic chronic rhinosinusitis with nasal polyps. *Allergy*.

[39] Glück J., Rymarczyk B., Jura-Szołtys E., Rogala B. (2019). Serum levels of interleukin 33 and its receptor ST2 in patients treated with subcutaneous allergen immunotherapy in intermittent allergic rhinitis. *Central-European journal of immunology.*.

