# Supplementary material for: Serum Soluble ST2 Correlated with Symptom Severity and Clinical Response of Sublingual Immunotherapy for House Dust Mite-Induced Allergic Rhinitis Patients
Source: Mediators Inflamm. 2021 May 30;2021:5576596. doi: 10.1155/2021/5576596 (PMC8181096; doi:10.1155/2021/5576596)
Supplement: Supplementary Materials — Information regarding allergen administration schedule in AR patients is described in Table S1. [file 5576596.f1.zip › Supplementary Description_5576596.docx]

Supplementary Description:

Information regarding Allergen administration schedule in AR patients was described in the Table S1.
