# Supplementary material for: Serum Soluble ST2 Correlated with Symptom Severity and Clinical Response of Sublingual Immunotherapy for House Dust Mite-Induced Allergic Rhinitis Patients
Source: Mediators Inflamm. 2021 May 30;2021:5576596. doi: 10.1155/2021/5576596 (PMC8181096; doi:10.1155/2021/5576596)
Supplement: Supplementary Materials — Information regarding allergen administration schedule in AR patients is described in Table S1. [file 5576596.f1.zip › Table S1.docx]

|  | Dose escalation phase | | |  | Dose maintaining phase | | |
| --- | --- | --- | --- | --- | --- | --- | --- |
| Days | Week 1  No.1 (drops) | Week 2  No.2 (drops) | Week 3  No.3 (drops) | Week 4-5  No.4 (drops) | | | ≥ week 6  No.5 (drops) |
| 1 | 1 | 1 | 1 | 3 drops daily | | | 2 drops daily for |
| 2 | 2 | 2 | 2 | for 2 weeks | | | maintenance |
| 3 | 3 | 3 | 3 |  | |  |  |
| 4 | 4 | 4 | 4 |  | |  |  |
| 5 | 6 | 6 | 6 |  | |  |  |
| 6 | 8 | 8 | 8 |  | |  |  |
| 7 | 10 | 10 | 10 |  | |  |  |

Table S1 Allergen administration schedule in AR patients

SLIT, sublingual immunotherapy; *Der f, Dermatophagoides farina;* AR, allergic rhinitis
